# Executive Functions in Neurodevelopmental Disorders: Comorbidity Overlaps Between Attention Deficit and Hyperactivity Disorder and Specific Learning Disorders

**DOI:** 10.3389/fnhum.2021.594234

**Published:** 2021-02-10

**Authors:** Giulia Crisci, Sara Caviola, Ramona Cardillo, Irene C. Mammarella

**Affiliations:** ^1^Department of Developmental and Social Psychology, University of Padua, Padua, Italy; ^2^School of Psychology, University of Leeds, Leeds, United Kingdom

**Keywords:** ADHD, SLD, comorbidity, neurodevelopmental disorders, executive functions

## Abstract

The present study examines the comorbidity between specific learning disorders (SLD) and attention deficit and hyperactivity disorder (ADHD) by comparing the neuropsychological profiles of children with and without this comorbidity. Ninety-seven schoolchildren from 8 to 14 years old were tested: a clinical sample of 49 children with ADHD (*n* = 18), SLD (*n* = 18) or SLD in comorbidity with ADHD (*n* = 13), and 48 typically-developing (TD) children matched for age and intelligence. Participants were administered tasks and questionnaires to confirm their initial diagnosis, and a battery of executive function (EF) tasks testing inhibition, shifting, and verbal and visuospatial updating. Using one-way ANOVAs, our results showed that all children in the clinical samples exhibited impairments on EF measures (inhibition and shifting tasks) when compared with TD children. A more specific pattern only emerged for the updating tasks. Only children with SLD had significant impairment in verbal updating, whereas children with ADHD, and those with SLD in comorbidity with ADHD, had the worst performance in visuospatial updating. The clinical and educational implications of these findings are discussed.

## Introduction

Neurodevelopmental disorders are mainly explained by a multiple cognitive deficit hypothesis (Willcutt et al., 2010), which emphasizes how clinical profiles are the outcome of complex interactions between several cognitive deficits and shared risk factors (that Pennington called the liabilities hypothesis; Pennington, 2006). These disorders are often characterized by the concomitant presence of more than one clinical condition, leading to the phenomenon of *comorbidity*. The extant research has clearly shown that various developmental problems tend to co-occur (Fawcett and Nicolson, 1995; Dewey and Wall, 1997; Piek et al., 1999), and that their symptoms may lie along a continuum of severity (Jensen et al., 2001; Kadesjö and Gillberg, 2001; Crawford et al., 2006). What is not clear, however, is whether children with these concomitant problems have two or more separate disorders or several symptoms associated with a single underlying condition. Comorbidity often means that developmental trajectories intersect for different disorders. Understanding these trajectories and how they intersect can shed light on their etiology and mutual interdependence (Pennington et al., 2005).

In particular, the comorbidity between attention deficit and hyperactivity disorder (ADHD) and specific learning disorders (SLD) has been widely studied, mainly because of their high prevalence (Lonergan et al., 2019; Astle and Fletcher-Watson, 2020), but also because they share several problems and symptoms. For example, when children have learning difficulties together with behavioral and attentional deficits, they exhibit symptoms that could indicate a learning disability and/or ADHD, raising issues in their diagnosis and treatment. The main challenge in this research field is to understand why these two disorders occur together, how they interact, and whether this comorbidity coincides with particular neuropsychological profiles.

### ADHD and SLD

ADHD is characterized by persistent inattention and/or hyperactivity traits interfering with normal development (DSM-5, American Psychiatric Association, 2013). The clinical picture of ADHD varies considerably, making it difficult to establish whether, in addition to inattention and hyperactivity, other traits should be considered as a part of the syndrome (Wåhlstedt et al., 2009). ADHD is one of the most often diagnosed disorders in childhood (Döpfner et al., 2015), although the prevalence estimates range from 0.2% to 34.5%, depending on the clinical and methodological approach used (Thomas et al., 2015; Reale and Bonati, 2018). Generally speaking, its prevalence is estimated worldwide at 5% in children under 18 years old (Polanczyk et al., 2007). Many children diagnosed with ADHD also have at least one other associated disorder (Tarver et al., 2014). Gillberg et al. (2004) report that the proportion ranges between 60% and 100%, depending on the studies considered (Ianes et al., 2009).

Although the neuropsychological profile of ADHD is heterogeneous, numerous studies indicate that it involves impairments in various executive function (EF) domains (Barkley et al., 1992; Pennington and Ozonoff, 1996; Sergeant et al., 2002). Reported findings are hardly conclusive, however, since the mean effect sizes range from small to moderate for EF measures, and not all children with ADHD show EF deficits (Willcutt et al., 2005), which can also be seen in typically-developing (TD) children (Vaidya et al., 2020), suggesting that none of these EF deficits is a necessary or sufficient explanation for the ADHD profile (Willcutt et al., 2003).

Another complex set of neurodevelopmental disorders are described by the umbrella term *specific learning disorders*/*disabilities* (SLD). According to the DSM-5, SLD is characterized by problems in academic skills, such as reading, writing, or arithmetic, which provide the foundations for other, more advanced academic learning (DSM-5, American Psychiatric Association, 2013). SLD mainly involve reading-related (dyslexia) and math-related (dyscalculia) disorders. The academic indicators of dyslexia include difficulties in word recognition and reading fluency (decoding skills). Children with dyscalculia may show problems in basic number processing, arithmetic facts, and calculation skills. SLD may also include deficits in reading comprehension, grammar, written expression, math reasoning, and problem-solving skills (Somale et al., 2016).

Like ADHD, so too for SLD, the prevalence estimates vary, mainly depending on the assessment procedures employed. The overall prevalence of SLD is thought to range from 5% to 15%, with 4% to 9% for dyslexia, and 3% to 7% for dyscalculia (Devine et al., 2013; Görker et al., 2017). On the one hand, considering domain-specific processes, dyslexia and dyscalculia seem to exhibit distinct cognitive profiles, with a phonological deficit in dyslexia (Coltheart, 2015), and a deficit in numerosity processing in dyscalculia (Landerl et al., 2009; Moll et al., 2015). On the other hand, when we consider domain-general cognitive processes, the two disorders share cognitive deficits—in working memory (WM), for instance (Schuchardt et al., 2008; Wilson et al., 2015; Moll et al., 2016; Peng and Fuchs, 2016; Toffalini et al., 2017; Mammarella et al., 2018b). These WM impairments might help to explain the co-occurrence of math and reading disorders in 30–70% of individuals diagnosed with SLD (Willcutt et al., 2013).

Moreover, also the comorbidity rate for SLD and ADHD ranges from 31% to 45% (DuPaul et al., 2013), but the incidence varies when specific academic domains are considered. The rate of comorbidity between reading-related deficits and ADHD ranges between 25% and 48% (Sadek, 2018), while it is estimated at between 11% and 30% for math-related deficits and ADHD (Capano et al., 2008). As comorbidity between ADHD and SLD is so common, the two different neuropsychological profiles sometimes seem to overlap (de Jong et al., 2009), but in other cases, a unique new problem seems to emerge (Bental and Tirosh, 2007).

### ADHD, SLD, and Executive Functions

In ADHD research, studies on the cognitive factors involved in SLD have generated mixed evidence, suggesting that although some deficits might be specifically related to SLD or ADHD, several factors might be shared (Pennington et al., 1984; Willcutt et al., 2010). Previous studies often showed that ADHD and SLD involve similar deficits in inhibition or planning (Marzocchi et al., 2002). Inhibition and planning are considered two of the most important EFs, which generally include several psychological processes, such as organizing, WM, attention, problem solving, verbal reasoning, cognitive flexibility, and monitoring (Diamond, 2013; Goldstein et al., 2014).

In the present study, we refer to Miyake’s model (Miyake et al., 2000), which identifies three basic EFs: (a) inhibition, or the ability to deliberately inhibit dominant, automatic, or imperious responses when required; (b) shifting (also called cognitive flexibility or switching), which is the ability to switch between tasks, operations or mental sets to adjust to changed priorities; and (c) updating, or the ability to update and monitor information in the WM, replacing old and no longer relevant information with more recent and relevant input, and translating instructions into action plans.

A huge amount of studies revealed the presence of inhibitory processes impairments in children with ADHD (Sonuga-Barke et al., 2002; Willcutt et al., 2005; Toll et al., 2011; Shimoni et al., 2012; Crosbie et al., 2013; Rajendran et al., 2013; Schreiber et al., 2014). Martinussen and Tannock (2006) also indicated WM as having an essential role in ADHD deficits. According to Alderson et al. (2010), major deficits can be seen in the central executive system, followed by visuospatial WM, and then verbal WM. Anomalies in visuospatial WM are thought to be among the most important deficits in the neuropsychological profile of children with ADHD (Prins et al., 2011). Finally, a few studies focused on shifting abilities in children with ADHD, with mixed results. Some studies found no shifting deficit (Biederman et al., 2007); some reported impaired shifting functions in terms of both accuracy and response times (O’Brien et al., 2010); and some only identified a lower accuracy (Holmes et al., 2010) or slower response times (Oades and Christiansen, 2008). These conflicting findings are probably due to the tasks chosen, which usually involve other EFs (Irwin et al., 2019).

On the other hand, the relationship between EFs and poor academic achievement is well documented (Mulder and Cragg, 2014). Children with SLD show deficits in central executive functioning (Landerl et al., 2004; Pickering, 2006), and particularly in WM (Mammarella et al., 2013; Moll et al., 2016; Peng and Fuchs, 2016). Verbal and visuospatial WM both seem to be related to the early acquisition of reading and math abilities (Passolunghi et al., 2008; Peng et al., 2018b). Later on, verbal WM is more implicated in reading performance and comprehension (Peng et al., 2018a), while visuospatial WM seems to be linked to more complex math achievement (Giofrè et al., 2014; Caviola et al., 2020). Moreover, mixed results have been reported for inhibition deficits in children with both reading and math disabilities, probably depending on the type of paradigm used (De Weerdt et al., 2013). Finally, meta-analyses by Yeniad et al. (2013) showed a substantial and significant association between shifting and math, as well as reading performance.

Despite an apparent overlap between the two disorders, few studies have directly compared the different neuropsychological profiles of children with ADHD, SLD, and comorbid ADHD + SLD. Willcutt et al. (2010) found individuals with reading disabilities more impaired than those with ADHD on measures of WM and rapid automated naming. Korkman and Pesonen (1994) reported that children with ADHD showed impairment in inhibition processes, while children with SLD tended to exhibit deficits in verbal aspects (e.g., verbal WM). Other researchers (Marzocchi et al., 2008; Faedda et al., 2019) found that their SLD group scored significantly higher than children with ADHD in all EFs.

As for the comorbidity issue, some researchers emphasized comorbidity as a qualitatively distinct condition (Pennington et al., 1993), showing that impairments relating to the two single disorders co-occurred in some cases (Willcutt et al., 2005; Kibby and Cohen, 2008; de Jong et al., 2009), while new deficits with a distinct cognitive deficit profile (called interactive effect) emerged in others (Bental and Tirosh, 2007). Moreover, further studies underscored the additive effect (i.e., the sum of the single cognitive deficit profiles) of two comorbid disorders (Seidman et al., 1995, 2001; Willcutt et al., 2013; Horowitz-Kraus, 2015). For instance, participants with comorbidity involving ADHD and SLD revealed worse EF deficits than those with ADHD alone (Seidman et al., 2001; Mattison and Mayes, 2012). To the best of our knowledge, however, only a few studies have compared two single deficits with the same two deficits in comorbidity, and such studies mainly considered comorbidity for ADHD and dyslexia. Some authors (Van De Voorde et al., 2010) found no differences in inhibition and WM tasks between cases with single deficits and those with a comorbid condition. Others (Bental and Tirosh, 2007) found more severe impairments in WM in comorbid than in single-deficit groups. As regards the WM task presentation format (verbal or visuospatial), Martinussen and Tannock (2006) found verbal WM performance to be worse in their groups with dyslexia (with or without ADHD), than in their group with ADHD alone. Kibby and Cohen (2008) found that the comorbid group performed worse in both verbal and visuospatial WM tasks than ADHD or dyslexia alone. In short, a definite conclusion has yet to be reached on this matter.

Taking into account the extant literature, to the best of our knowledge, previous studies rarely compared EF profile in children with a clinical diagnosis of ADHD and SLD in comorbidity, with children who had either ADHD or SLD (with both reading and math impairments), despite some studies highlighted the importance of EF as potential shared risk factor between SLD and ADHD (Pennington et al., 2005; Pennington, 2006). This reveals a potential methodological bias in our understanding of the role of specific deficits in EF domains in these disorders. Astle and Fletcher-Watson (2020) suggested that this was because studies often used strict exclusion criteria that excluded children with co-occurring difficulties (Willcutt et al., 2001; Toplak et al., 2005). Since comorbidity is common in neurodevelopmental disorders, rather than an exception (Gillberg, 2010), we need to include a comorbid group (ADHD + SLD) in our efforts to understand the neuropsychological differences between the two disorders.

### The Present Study

As previous studies showed that children with ADHD and SLD may both have specific EF deficits (Willcutt et al., 2010; Schreiber et al., 2014; Peng and Fuchs, 2016; Faedda et al., 2019), we analyzed EF profile to reveal potential differences in the profiles associated with ADHD and SLD considered separately, but also in comorbidity (ADHD + SLD). As mentioned earlier, no systematic studies in EF have directly compared children with a clinical diagnosis of ADHD, SLD, and ADHD + SLD.

We, therefore, assessed different EF components in four groups of children: children with ADHD; children with SLD; children with ADHD + SLD; and a control group of TD children. In our study measures of inhibition, shifting, and updating (verbal and visuospatial) were administered. Samples of children with a clinical diagnosis were matched with TD children for chronological age and intelligence level. Our main aims were to investigate specific impairments in EF domains in the clinical groups and to test the potential additive effect of the comorbidity between ADHD + SLD.

Based on previous studies, we expected all children in the clinical groups (ADHD, SLD, and ADHD + SLD) to show EF impairments (Hari and Renvall, 2001; Sergeant et al., 2002; Martel et al., 2007; Bull et al., 2008; Barkley, 2011) compared to TD children. We expected children with ADHD to have significant impairments in all EF measures compared with TD children, except for updating tasks, where we expected the ADHD group’s performance to differ depending on the presentation format (verbal vs. visuospatial): the ADHD group was expected to perform less well than the TD group in the visuospatial task, but not in verbal one (Prins et al., 2011). Based on previous studies, the SLD group was expected to perform less well than the TD group in terms of inhibition (De Weerdt et al., 2013), shifting (Yeniad et al., 2013), and both verbal and visuospatial updating (Peng et al., 2018a; Caviola et al., 2020).

We expected that children with ADHD and SLD had difficulties in both inhibition and shifting, with specific WM differences, according to the presentation format (Willcutt et al., 2001; de Jong et al., 2009). Children with ADHD were expected to perform worse than children with SLD in visuospatial updating (de Jong et al., 2009). In contrast, children with SLD were expected to show more impairments in verbal updating (Kibby and Cohen, 2008).

Considering the few, inconsistent studies in the literature, we might expect several cognitive profiles in children with comorbid ADHD and SLD compared with those with either ADHD or SLD. Children with comorbid ADHD + SLD could have a more significantly impaired neuropsychological profile than those with a single disorder (Seidman et al., 2001; Mattison and Mayes, 2012), in line with an additive effect of the two disorders together (Willcutt et al., 2013). We might also expect children with comorbid ADHD + SLD to have a worse EF performance than those with a single neurodevelopmental disorder (either ADHD or SLD; Fernández-Andrés et al., 2019), pointing to the co-occurrence of the symptoms of the two clinical conditions rather than a third, separate disorder with a qualitatively different cognitive subtype.

**Table 1:** Characteristics of the groups: means (M) and standard deviations (SD) for group selection measures.

		ADHD (*n* = 18) *M* (SD)	SLD (*n* = 18) *M* (SD)	ADHD + SLD (*n* = 13) *M* (SD)	TD (*n* = 48) *M* (SD)	ANOVAs *F*_(3,93)_	*p*	*Adjusted* *R*^2^	*Post-hoc*
Age (in months)		123.11 (20.48)	136.83 (17.67)	134.15 (24.70)	133.08 (20.15)	1.56	0.20	0.02	
IQ		108.50 (8.95)	109.39 (8.86)	107.92 (13.27)	110.56 (8.66)	0.39	0.76	0.02	
CPRS-R:S (*T*-score)	Oppositive	66.00 (14.75)	52.17 (11.09)	58.62 (12.72)	50.40 (10.26)	8.56	<0.001	0.19	ADHD, ADHD+SLD > TD; ADHD > SLD
	Inattention	75.06 (12.99)	57.39 (8.3)	64.46 (10.03)	48.27 (8.74)	35.97	<0.001	0.52	ADHD > ADHD+SLD > SLD > TD
	Hyperactivity	62.78 (13.86)	45.17 (3.37)	59.92 (13.91)	46.69 (7.08)	19.18	<0.001	0.36	ADHD, ADHD+SLD > SLD, TD
	ADHD index	76.11 (12.14)	56.94 (8.43)	66.23 (12.20)	48.48 (8.25)	39.69	<0.001	0.55	ADHD > ADHD+SLD > SLD > TD
DDE-2 (*z*-score)	Words, syll/sec	−0.28 (0.95)	−1.79 (1.07)	−1.41 (1.35)	−0.10 (1.23)	11.69	<0.001	0.25	TD, ADHD > SLD, ADHD+SLD
	Words, errors	0.39 (1.32)	1.23 (1.23)	2.83 (3.76)	−0.11 (0.97)	11.12	<0.001	0.24	ADHD+SLD > SLD > TD; ADHD+SLD > ADHD
	Pseudo-words syll/sec	−0.27 (0.69)	−1.39 (0.92)	−1.22 (1.17)	−0.18 (1.08)	8.72	<0.001	0.19	TD, ADHD > SLD, ADHD+SLD
	Pseudo-words, errors	−0.17 (0.72)	1.17 (1.54)	0.75 (1.53)	−0.44 (0.86)	11.47	<0.001	0.25	SLD, ADHD+SLD > TD, ADHD
DDE-2 (*z*-score)	Homophones not homographs	0.96 (1.20)	1.92 (1.27)	2.94 (1.56)	0.82 (0.95)	13.69	<0.001	0.28	ADHD+SLD > SLD > TD, ADHD
AC-MT (*z*-score)	Mental C–errors	0.14 (1.25)	0.41 (1.11)	0.73 (1.59)	−0.53 (0.81)	6.77	<0.001	0.15	ADHD, SLD, ADHD+SLD > TD
	Mental C–time	0.25 (0.95)	1.13 (1.07)	0.47 (1.25)	−0.15 (1.00)	6.86	<0.001	0.15	ADHD+SLD, SLD > TD
	Written C–errors	−0.24 (0.53)	1.06 (1.10)	1.37 (1.28)	−0.14 (0.88)	15.15	<0.001	0.31	SLD, ADHD+SLD > TD, ADHD
	Written C–time	0.18 (1.12)	1.61 (1.31)	1.45 (2.14)	0.46 (1.30)	4.95	0.003	0.11	TD, ADHD > SLD, ADHD+SLD
	Transcoding	−0.05 (1.24)	1.52 (2.28)	1.38 (2.08)	−0.24 (0.78)	9.26	<0.001	0.21	SLD, ADHD+SLD > TD, ADHD
	Fact retrieval	0.19 (1.7)	1.52 (1.03)	0.64 (1.50)	−0.71 (0.84)	17.28	<0.001	0.34	SLD > ADHD+SLD, ADHD > TD
AC-FL (raw score)		25.56 (13.3)	22.64 (9.02)	20.15 (11.39)	34.71 (12.48)	7.98	<0.001	0.18	TD > ADHD, SLD, ADHD+SLD

*Note: ADHD, attention deficit and hyperactivity disorder; SLD, specific learning disabilities; ADHD+SLD, ADHD and SLD in comorbidity; TD, typical development; IQ, intelligence quotient; CPRS-R:S, Conners’ Parent Rating Scale; DDE-2, Battery for assessing developmental dyslexia and dysorthographia–2; Mental C, mental calculation; Written C, written calculation*.

## Materials and Methods

### Participants

The total sample consisted of 97 children, 66 males, and 31 females, aged between 8 and 14 years (*M* = 11, SD = 1.73). Children with a clinical diagnosis of ADHD, SLD, or ADHD + SLD were recruited at the child and adolescent neuropsychiatry services. TD children were enrolled at primary and secondary schools. The children in the clinical groups had already been independently diagnosed according to the DSM-5 (American Psychiatric Association, 2013), based on comprehensive assessments reported in their medical records. All children in the SLD group had been clinically diagnosed as cases of SLD, with major impairments in both math and reading abilities. Table 1 summarizes the general characteristics of the four groups.

All participants were native Italian speakers, and none had any diagnosed neurological conditions. Exclusion criteria for all participants were: a history or concurrent diagnosis of other neurodevelopmental disorders; a history of neurological problems; current use of medication; medical illness requiring immediate treatment; psychological treatments in progress; or a certified intelligence quotient (IQ) below 80 (Table 1). The clinical groups consisted of: 18 children with ADHD (*M* = 123.11 months, SD = 20.48); 18 with SLD (*M* = 136.83 months, SD = 17.67); and 13 with ADHD + SLD (*M* = 134.15 months, SD = 24.7). They were matched with 48 TD children (*M* = 133.08 months, SD = 20.15) for chronological age (*F*_(3,93)_ = 1.56, *p* = 0.20, *AdjustedR^2^* = 0.02), gender (*χ*^2^_(df = 3)_ = 5.16, *p* = 0.16, *Cramer-V* = 0.231), and FSIQ^1^ (*F*_(3,93)_ = 0.39, *p* = 0.76, *AdjustedR^2^* = 0.02).

For the study, all diagnoses were confirmed by assessing ADHD symptoms and learning difficulties as explained below in the “Group Selection Measures” section.

The Research Ethics Committee of the University of Padua approved the study.

### Materials

#### Group Selection Measures

##### Conners Rating Scale-Revised

CPRS R:S (Conners, 1997). This parent-report was used in the clinical evaluation of ADHD to identify and measure the intensity of inattention, hyperactivity, and impulsivity traits. It covers the criteria listed in the Diagnostic and Statistical Manual of Mental Disorders [4th Edition text revision (DSM-IV-TR); American Psychiatric Association, 2000] and oppositional traits that are often seen in children with ADHD. It took under 10 min to complete. The parent’s form, consisting of 27 items, was used in this study to confirm the presence of ADHD symptoms. A parent-rated how much the symptoms described had been typical of their child’s behavior during the previous month using a 4-point Likert scale from 0 (not true at all) to 3 (very true). Cronbach’s alpha ranged from 0.86 to 0.94 (Maruish, 2004).

##### Reading Tasks

DDE-2 (Sartori et al., 2007). Children’s reading skills were measured with two different tasks that involved reading lists of words and pseudo-words. The first consisted of four lists of 28 words each, including high-frequency words (i.e., man, morning) and low-frequency words (i.e., prowess, globule) of two to four syllables. In the pseudo-words task, there were three lists of 16 made-up words each. Participants were asked to read each word out loud as quickly and accurately as possible. The experimenter recorded the time spent on each list, and scored the reading errors (letter substitutions, omissions, position changes, or additions), scoring no more than one error point for any given word. Self-corrections were not counted as errors. Reading performance was measured in terms of: (1) reading speed, i.e., the number of syllables read per second, expressed as the total reading time for each list; and (2) reading errors, i.e., the total number of words misread. Reliability varies from *r* = 0.74 to *r* = 0.96 (Di Brina et al., 2018).

##### Writing Task

DDE-2 (Sartori et al., 2007). Children’s spelling competence was tested with a “homophones-not-homographs test.” They were asked to write a list of sentences read aloud by the experimenter that contained some words with the same pronunciation but different spelling. The appropriate spelling depended on the word’s meaning drawn from the overall context (i.e., “flower” and “flour”). Only errors relating to this type of word were considered, scoring no more than one error point for any given word. Reliability varies from *r* = 0.74 to *r* = 0.96 (Di Brina et al., 2018).

##### Arithmetic Task

AC-MT 6-11; 11-14 (Cornoldi and Cazzola, 2003; Cornoldi et al., 2012). Math competencies were assessed with the AC-MT battery, with the age-appropriate subtests. For the present study, children were administered the individual part of the AC-MT battery, consisting of mental and written calculation, transcoding, and fact retrieval tasks. Mental and written calculations involved additions, subtractions, multiplications, and divisions appropriate for the participant’s age and school level. The transcoding and fact retrieval tasks assessed their basic numerical knowledge. For both mental and written calculations, problems were administered verbally only once, and primary-school children were allowed up to 30 s (mental calculation) or 60 s (written calculation) to answer them, while middle-school children were allowed 60 s for both types of calculation. The number of errors and the time taken to respond were recorded. In the transcoding task, the experimenter read one number at a time aloud, and only once. The fact retrieval task involved children directly retrieving simple solutions to arithmetical problems within 5 s. For both these tasks, only the number of errors was considered. Test-retest coefficients range from *r* = 0.70 to *r* = 0.79 for primary-school children, and from *r* = 0.72 to *r* = 0.83 for secondary-school children (Hill et al., 2016).

##### Math Fluency Task

AC-FL (Caviola et al., 2016). In this task, the children were asked to solve three sets of calculations (additions, subtractions, and multiplications). They had 2 min to complete each set of problems as quickly and accurately as possible. Each set contained 24 complex problems involving two- or three-digit numbers. The task implicitly assessed children’s calculation strategies. The total number of correct solutions was recorded. Cronbach’s α was 0.89, 0.90, and 0.82 for additions, subtractions, and multiplications, respectively (Caviola et al., 2016).

#### Executive Function Tasks

##### Inhibition and Shifting

NEPSY II (Korkman et al., 2007). This task assesses the ability to inhibit automatic responses in favor of novel answers, and the ability to switch automatic responses. The children were shown a series of black and white shapes or arrows pointing in different directions. The task involved two conditions: (a) an inhibition condition, in which participants had to name the opposite shapes (i.e., if they saw a square the children should say “circle” and vice versa) or arrow directions (i.e., if the arrow was pointing upwards they should say downwards, and vice versa) as rapidly and accurately as possible; and (b) a shifting condition, in which they had to name shapes (or directions of arrows) differently depending on their color (i.e., if the shape or arrow was black, they had to say what they were seeing; if it was white, they had to name the opposite shape or direction). Response times and errors were recorded. According to the manual, response times were first converted into standard scores, and errors were converted into percentiles. Then the two scores obtained were converted into a single standardized “combined score” that took both parameters into account. Test-retest reliability ranges between *r* = 0.79 to *r* = 0.82 for the inhibition condition, and between 0.75 and 0.93 for the shifting condition (Brooks et al., 2009).

##### Verbal and Visuospatial Updating

Two updating tasks were devised with different types of stimuli, verbal in one and visuospatial in the other. Both tests, administered using E-prime (Schneider and Zuccoloto, 2007) and a laptop computer with a 15-inch LCD screen, were characterized by four levels of difficulty depending on the increased number of target categories. Each level consisted of two items in which the memory span required stayed the same. The children were asked to recall the last verbal stimulus or its last positions belonging to *target categories* (among 2–5) shown on the computer screen. A detailed description of both verbal and visuospatial updating is reported in the Supplementary Materials.

Accuracy in both verbal and visuospatial tasks was considered, based on the proportion of items correctly remembered out of the total words or positions to remember. Cronbach’s α based on the current sample was 0.71 for verbal updating and 0.76 for visuospatial updating.

### Procedure

After obtaining the written consent of children’s parents to their participation in the study, the children were tested during two different sessions in a quiet room outside their classrooms (for TD children) or at the Child Neuropsychiatry Department of the hospital to which they referred for their diagnosis (for children in the clinical groups). At the same time, parents completed a rating scale to assess their children’s ADHD symptoms.

Participants completed both the group selection measures and the cognitive tasks, administered in a counterbalanced order, during two individual sessions lasting approximately 1 h each. Instructions were given for each task, and participants practiced with each task before starting the experiment. All experimental tasks were preceded by two practice trials. For the computer-based tasks, the children sat in front of the computer screen and the experimenter sat on the child’s right to present the tasks.

## Results

### Data Analysis

Data analyses were conducted using R (RC Team, 2015). One-way ANOVAs were run for the group selection measures and the inhibition task, to examine the differences between groups.

The analyses were run in two stages. In the first, Group was included as an independent variable. In the second, to answer the question of whether or not the comorbid group has an additive profile, the same analyses as in the first stage were run, with the presence of ADHD (no/yes) and SLD (no/yes) as factors^2^.

The Akaike information criterion (*AIC*, Akaike, 1974) was also taken into consideration for each of these models. It provided the best description of the relationships between the variables (Bentler, 1990; Schermelleh-Engel et al., 2003). Graphical effects were obtained using the “effects” package (Fox, 2003). The Supplementary Results contain detailed analyses of the updating tasks by span level.

The updating tasks (both verbal and visuospatial) allowed us to collect accurate data for each item from each participant. Generalized mixed-effects models were used (Baayen et al., 2008; Jaeger, 2008) and a “binomial” function family, using the “lme4” package (Bates et al., 2015). Participants were included as random effects. This latter analysis is extensively described in the Supplementary Results section.

### Group Selection

In the first phase, the Conners’ Parent Rating Scale-Revised, Short-Form (CPRS-R:S, Conners, 1997) was used to confirm their children’s inattention and/or hyperactivity symptoms, and T-scores of 65 or more were required for inclusion in the ADHD group. To be assigned to the SLD group, children were required to show an impaired performance (>−2 SD) in at least one domain of academic achievement: reading (DDE-2; Sartori et al., 2007); spelling (DDE-2; Sartori et al., 2007); or math (AC-MT 6-11, Cornoldi et al., 2012; AC-MT 11–14, Cornoldi and Cazzola, 2003; AC-FL, Caviola et al., 2016). Confirmation of ADHD in comorbidity with SLD (ADHD + SLD group) required an impaired performance (>−2 SD) in at least one domain of academic achievement and a T-score of 65 or higher in the CPRS-R:S indexes for Inattention or ADHD.

As shown in Table 1; the group profiles were confirmed. Children with ADHD (with or without SLD) had significantly higher scores in CPRS-R indexes than those with TD and SLD, showing at least two clinically significant indices. Children with SLD (with or without ADHD) were more impaired in reading and writing than TD and ADHD. As for math abilities, all clinical groups performed significantly worse than children with TD. The ADHD group had a significantly better performance than SLD and ADHD + SLD in both transcoding and written calculation.

### Executive Functions

#### Inhibition

**Figure 1:**
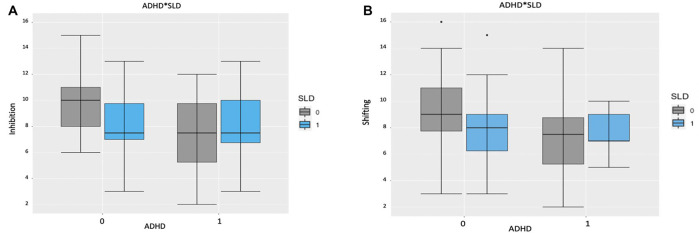
**(A)** Boxplots representing the composite score by attention deficit and hyperactivity disorder (ADHD) and specific learning disorders (SLD) in the Inhibition task. The central mark is the median. The edges of the box are the 25th and 75th percentiles. The whiskers are the interquartile range. Note: 0 = absent; 1 = present. **(B)** Boxplots representing the composite score by ADHD and SLD in the Switching task. The central mark is the median. The edges of the box are the 25th and 75th percentiles. The whiskers are in the interquartile range and the symbols are outliers. Note: 0 = absent; 1 = present.

Table 2 sums up the descriptive statistics by group (ADHD, SLD, ADHD + SLD, and TD) in the inhibition and shifting conditions. In the first stage, a main effect of Group emerged in both inhibition (*F*_(3,93)_ = 6.80, *p* < 0.001, *AdjustedR^2^* = 0.15), and shifting (*F*_(3,93)_ = 3.27, *p* = 0.025, *AdjustedR^2^* = 0.07). For both conditions, children with a clinical diagnosis performed significantly worse than TD children (inhibition: ADHD: *p* < 0.001, *Cohen’s d* = 0.96; SLD: *p* = 0.01, *Cohen’s d* = 0.83; ADHD + SLD: *p* = 0.002, *Cohen’s d* = 0.89; shifting: ADHD: *p* = 0.01, *Cohen’s d* = 0.66; SLD: *p* = 0.05, *Cohen’s d* = 0.57; ADHD + SLD: *p* = 0.056, *Cohen’s d* = 0.73). No other differences emerged between the groups. In the second stage, the same analyses were run using the presence of ADHD and SLD as factors. In the inhibition task, a main effect of ADHD emerged (*F*_(1,95)_ = 11.04, *p* = 0.001, full model: *AIC* = 451.96, model without ADHD *AIC* = 460.74) and SLD (*F*_(1,95)_ = 4.30, *p* = 0.04, model without SLD *AIC* = 454.30). As shown in Figure 1A, the interaction was not significant (*F*_(1,93)_ = 2.40, *p* = 0.12, model with interaction *AIC* = 451.49).

**Table 2:** Measures of executive functions: means (M) and standard deviations (SD) by group.

		ADHD (*n* = 18) *M* (SD)	SLD (*n* = 18) *M* (SD)	ADHD+SLD (*n* = 13) *M* (SD)	TD (*n* = 48) *M* (SD)
Inhibition	Combined	7.33 (3.03)	8.00 (2.28)	7.31 (3.33)	9.73 (1.85)
Shifting	Combined	7.39 (3.11)	7.83 (2.85)	7.69 (1.65)	9.31 (2.65)
Verbal updating	Accuracy	0.65 (0.11)	0.56 (0.13)	0.63 (0.12)	0.66 (0.11)
Visuospatial updating	Accuracy	0.57 (0.19)	0.71 (0.09)	0.58 (0.17)	0.69 (0.18)

*Note: ADHD, attention deficit and hyperactivity disorder; SLD, specific learning disabilities; ADHD+SLD, ADHD and SLD in comorbidity; TD, typical development*.

In the shifting task, a main effect emerged for ADHD (*F*_(1,95)_ = 4.60, *p* = 0.03, full model: *AIC* = 472.32, model without ADHD *AIC* = 474.96), but not for SLD (*F*_(1,95)_ = 1.94, *p* = 0.16, model without SLD *AIC* = 472.31). As shown in Figure 1B, the interaction was not significant (*F*_(1,93)_ = 2.12, *p* = 0.15 model with interaction *AIC* = 472.13).

### Verbal and Visuospatial Updating

Table 2 sums up the descriptive statistics by group (ADHD, SLD, ADHD + SLD, and TD) in the Verbal Updating and Visuospatial Updating. In the first stage, a main effect of Group emerged in Verbal updating (*F*_(3,93)_ = 3.40, *p* = 0.02, *AdjustedR^2^* = 0.07), as children with a clinical diagnosis of SLD performed significantly worse than children with TD or ADHD (respectively: *p* = 0.003, *Cohen’s d* = 0.83; and *p* = 0.01, *Cohen’s d* = 0.83). There was also a main effect of Group in Visuospatial updating (*F*_(3,93)_ = 3.59, *p* = 0.02, *AdjustedR*^2^ = 0.07), as children with ADHD and ADHD + SLD performed significantly worse than the TD or SLD groups (for TD: *p* = 0.01, *Cohen’s d* = 0.65 and *p* = 0.04, *Cohen’s d* = 0.63, respectively; for SLD: *p* = 0.02, *Cohen’s d* = 0.94 and *p* = 0.05, *Cohen’s d* = 0.96). No other differences emerged between the groups.

**Figure 2:**
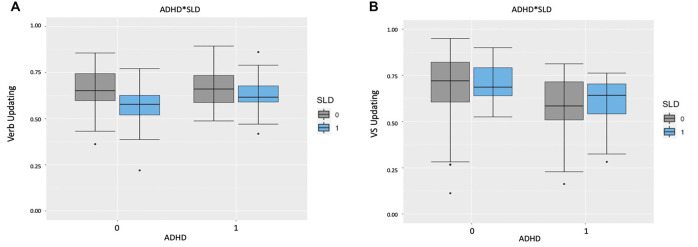
**(A)** Boxplots representing the accuracy score by ADHD and SLD in the Verbal Updating Task. The central mark is the median. The edges of the box are the 25th and 75th percentiles. The whiskers are in the interquartile range and the symbols are outliers. Note: 0 = absent; 1 = present. **(B)** Boxplots representing the Accuracy Score by ADHD and SLD in the Visuospatial Updating Task. The central mark is the median. The edges of the box are the 25th and 75th percentiles. The whiskers are in the interquartile range and the symbols are outliers. Note: VS Updating = Visuospatial Updating; 0 = absent; 1 = present.

In the second stage, using ADHD and SLD as factors, a main effect on the Verbal updating task emerged for SLD (*F*_(1,95)_ = 7.90, *p* = 0.006, full model: *AIC* = 133.77, model without SLD *AIC* = 139.94), but not for ADHD (*F*_(1,95)_ = 1.04, *p* = 0.31, model without ADHD *AIC* = 140.69). As shown in Figure 2A, the interaction was not significant (*F*_(1,93)_ = 1.85, *p* = 0.18, model with interaction *AIC* = 133.68). In the Visuospatial updating task, there was a main effect of ADHD (*F*_(1,95)_ = 10.87, *p* = 0.001, full model: *AIC* = 55.64, model without ADHD *AIC* = 64.26), but not of SLD (*F*_(1,95)_ = 0.15, *p* = 0.70, model without SLD *AIC* = 66.11). Here again, the interaction was not significant (*F*_(1,93)_ = 0.004, *p* = 0.95, model with interaction *AIC* = 62.27), as shown in Figure 2B.

Finally, in the mixed-model analysis (extensively reported in the Supplementary Results) no main effect of the group emerged in the Verbal updating task. Instead, there was a significant main effect of Span (*χ*^2^_(3)_ = 184.52, *p* < 0.001, model without Span: *AIC* = 1,993.9). No significant interaction between Group and Span emerged. In the Visuospatial updating task, there was a main effect of Group (*χ*^2^_(3)_ = 10.55, *p* = 0.01, full model: *AIC* = 2,013.5, model without Group: *AIC* = 2,018) and Span (*χ*^2^_(3)_ = 100.11, *p* < 0.001, model without Span: *AIC* = 2,107.6). The interaction between Group and Span was also significant (*χ*^2^_(9)_ = 33.63, *p* < 0.001, model with Interaction: *AIC* = 1,997.8).

## Discussion

The main aim of our study was to examine the specific neuropsychological profiles of children with a clinical diagnosis of either ADHD or SLD—with major impairment in both reading and math, or both in comorbidity (ADHD + SLD), by comparison with TD children. We were particularly interested in understanding whether the EFs profiles of four groups differed and whether the comorbid group (ADHD + SLD) showed an additive (i.e., the sum of the deficits in the isolated groups) or rather an interactive effect (i.e., a distinct deficit profile). Children in the clinical groups had been previously diagnosed at centers specialized in neurodevelopmental disorders. In the first part of the assessment, all their diagnoses had been confirmed through specific questionnaires for parents and appropriate academic achievement tests.

To test potential differences in EFs profiles, children with a clinical diagnosis of ADHD, SLD, and comorbid ADHD + SLD were compared with TD children on measures of inhibition, shifting, and updating (verbal and visuospatial). In our analyses, we first compared our groups considering EF measures separately. Then, we ran the same analyses considering the presence of ADHD (no/yes) and/or SLD (no/yes) as factors to see whether the comorbid group reveals an additive profile. Finally, mixed-effects models were used to analyze in detail performances at different span levels for the updating tasks.

In the group comparisons, our findings showed that all clinical groups performed worse than the TD group, and no differences emerged between any of the clinical groups on measures of inhibition and shifting. A more specific pattern emerged when the groups were compared on updating measures. Children with SLD performed less well than the other groups in the verbal task, while the groups with ADHD or ADHD + SLD performed less well than either the SLD or the TD groups in the visuospatial task. This would contradict the idea of an additive effect of the two disorders combined (Seidman et al., 1995, 2001; Willcutt et al., 2013; Horowitz-Kraus, 2015). The pattern was slightly different when we considered the presence or absence of symptoms of SLD or ADHD: the effects of both SLD and ADHD could be seen in the inhibition task, but only those of ADHD in the shifting task. The effect of SLD was apparent for verbal updating and that of ADHD for visuospatial updating. Notably, from a qualitative perspective, children with ADHD + SLD were not more severely impaired than those with either ADHD or SLD alone. This would contradict the interactive hypothesis that children with several problems in comorbidity exhibit a qualitatively distinct condition (Pennington et al., 1993). Finally, by considering group performances at different span levels, a specific pattern emerged in the visuospatial updating task. Children with ADHD performed significantly worse on Span level 3 then showed a slight improvement on level 4, whereas the other groups had a more linear worsening performance with longer spans. Our results can be explained by altered motivational processes in ADHD (Sagvolden et al., 2005), or the children’s inability to regulate their state of activation (Kuntsi and Klein, 2011).

The novelty of our investigation lies in that we compared these clinical groups with one another, as well as with a TD group, as previously reported. The results underlined that EFs are similarly compromised in all clinical groups, pointing to a comorbidity explanation based on a domain-general cognitive level. In particular, EF impairments, are not enough to differentiate between ADHD and SLD (Stern and Morris, 2013), shedding further light on the importance of comparisons across disorders and studies on comorbid conditions. Although ADHD is often associated with EF deficits (Barkley et al., 1992; Pennington and Ozonoff, 1996; Sergeant et al., 2003), this association did not seem sufficient to consider EF as core-deficits of the disorder (Willcutt et al., 2003), and impairments in inhibition (Booth et al., 2010; Mammarella et al., 2018a) and shifting (Van der Sluis et al., 2007; Andersson, 2008) have also been observed in children with SLD.

It is worth noting that SLD involves specific difficulties relating to achievement, particularly in reading (dyslexia) and math (dyscalculia). Dyslexia and dyscalculia seem to involve distinct cognitive profiles in terms of domain-specific processes (mainly phonological deficits for the former, and number processing deficits for the latter), but similar domain-general cognitive processes (particularly concerning WM). Domain-general cognitive processes like WM may therefore substantially overlap between dyslexia and dyscalculia (Peters and Ansari, 2019). In the present study, our SLD group consisted of children with major impairments in both math and reading abilities, unfortunately making it impossible to separately analyze the influence of reading or math. Our groups of children with either SLD or ADHD showed more specific patterns of results when looking at domain-general processes, linked to the presentation format of the WM tasks. In agreement with previous studies (Willcutt et al., 2001; de Jong et al., 2009), when verbal and visuospatial WM updating were compared, specific differences emerged between ADHD and SLD. Children with ADHD (with or without SLD) performed significantly worse than children with SLD in visuospatial updating (Kibby and Cohen, 2008; de Jong et al., 2009). In contrast, children with SLD were significantly more impaired than children with ADHD in verbal updating (Korkman and Pesonen, 1994; Willcutt et al., 2001; Kibby and Cohen, 2008). Our results thus suggest that the presentation format of an updating task (i.e., verbal or visuospatial), rather than the cognitive task *per se*, may be useful for distinguishing between ADHD and SLD.

As concerns comorbid ADHD + SLD, our data would support the claim that ADHD + SLD is not a third, separate disorder with a specific pattern of EF impairments since we could find no specific profile distinctive of children with both conditions. Thus, we could not rule out the possibility of ADHD and SLD shared the same biological and environmental risk factors, increasing the likelihood of their co-occurrence and supporting the correlated liabilities hypothesis (Pennington et al., 2005; Pennington, 2006).

Although our study produced some interesting findings, our results should be considered explorative because it has some limitations. First of all, the sample size was small and the children in the SLD group had significant impairments in both reading and math, which prevented us from analyzing their influence separately. Second, the SLD group also had some attention-related problems, though they were not clinically relevant, and some differences in achievement emerged between groups of ADHD and typical development and SLD and ADHD + SLD. It is worth emphasizing that the children in our clinical groups had previously received a clinical diagnosis, and the heterogeneity of our sample’s difficulties was typical of neurodevelopmental disorders and the impairments were not fulfilling criteria for different diagnoses. Another limitation of our study lies in that we only administered a limited set of EF tasks, without differentiating between verbal and visuospatial tasks for inhibition and shifting. We chose these particular tasks because the procedure was already long and hard, particularly for children with ADHD, and because they reflected our theoretical background (Miyake et al., 2000). Finally, our group with comorbid ADHD and SLD was smaller than the other two. This was because, we paid more attention to confirming the comorbid condition (ADHD + SLD, without any other comorbidities). Further research might replicate our methodology but increasing the numerosity of the clinical samples and including other cognitive tests.

Even with the above-mentioned limitations, our study has some important clinical implications. Understanding the specific type of interaction, the similarities, and differences between ADHD and SLD, and the combination of the two is fundamental to our ability to assess and treat all three conditions. The DSM-5 (American Psychiatric Association, 2013) made an important effort to operationalize the concept of a dimensional approach to neurodevelopmental disorders, but some issues persist (Pham and Riviere, 2015). We agree with previous studies that a neuropsychological assessment is not enough to convey a diagnosis (for further details, see Pham and Riviere, 2015). As, we have reported, children with ADHD can have learning difficulties, and children with SLD can have attention deficits, and our group of children with both disorders did not have a specific domain-general cognitive profile. Neuropsychological impairments and learning difficulties are not as uniquely associated with these disorders as was earlier supposed (Nigg and Huang-Pollock, 2003; Happé et al., 2006). Clinicians should therefore bear in mind the kinds of challenges they may encounter in the assessment process and the differential diagnosis. It is good practice not to focus on seeking specific neuropsychological deficits associated with a potential disorder, but rather to assess a child’s abilities as a whole, to identify particular strengths and weaknesses.

To conclude, it is important to emphasize that no important differences emerged from our study between the clinical conditions considered as regards the children’s EF impairments. All three clinical groups were significantly impaired by comparison with TD children. However, a more specific pattern emerged for the WM updating, in which verbal and visuospatial presentation format seems to better differentiate the SLD and ADHD profiles. Nevertheless, further studies are needed to confirm our findings.

## Data Availability Statement

The raw data supporting the conclusions of this article will be made available by the authors, without undue reservation.

## Ethics Statement

The studies involving human participants were reviewed and approved by Psychology Research Ethics Committee of the University of Padua. Written informed consent to participate in this study was provided by the participants’ legal guardian/next of kin.

## Author Contributions

SC and IM developed the study concept. Testing was performed by GC. RC and GC performed the data analysis. GC and SC drafted the manuscript and IM provided revisions. All authors contributed to the article and approved the submitted version.

## Conflict of Interest

The authors declare that the research was conducted in the absence of any commercial or financial relationships that could be construed as a potential conflict of interest.

## Footnotes

^1^All children in the clinical sample had already been diagnosed after a comprehensive clinical assessment that included the whole WISC IV battery (Wechsler, 2003), but only their full-scale IQ was made available to us by the clinical centers involved.

^2^Additional analyses were run, controlling for the role of attentional difficulties derived from the CPRS: R-S. The results revealed no group differences for inhibition, switching, or visuospatial updating tasks after controlling for attentional difficulties. A slight difference emerged in the verbal updating task, with the SLD group performing worse than the other clinical groups (with ADHD or ADHD + SLD).
