# Supplementary material for: Executive Functions in Neurodevelopmental Disorders: Comorbidity Overlaps Between Attention Deficit and Hyperactivity Disorder and Specific Learning Disorders
Source: Front Hum Neurosci. 2021 Feb 10;15:594234. doi: 10.3389/fnhum.2021.594234 (PMC7958764; doi:10.3389/fnhum.2021.594234)

Supplementary Materials

Verbal updating task

This task involved 8 word lists containing from 6 to 12 words each, verbally presented using a computer, at a rate of 1 word per second. Each list included both target and distractor words. Only high-frequency words from the following categories were used: body parts, clothes, jobs, food, vegetables, fruit, animals, months, cities, and means of transport. Words belonging to the same superordinate category (i.e. fruit, vegetable and food) were never presented together.

Participants were presented with 2 word lists for each level of increasing difficulty deriving from the increasing number of categories to monitor while listening. The number of target categories increased from 2 to 5, corresponding to span levels from 2 to 5. For example, a 6-word list consisted of 4 words from two target categories and 2 words from a single non-target category.

The task involved recalling, for each list, the last word heard that belonged to a given target category. The target categories remained visible at the center of the screen throughout the presentation of the word lists. Below is an example of the task, showing the lower level of difficulty, i.e. a 6-word list, with two target categories (span 2; Figure S1).

Figure S1

Example of the Verbal updating task showing a trial at span level 2.

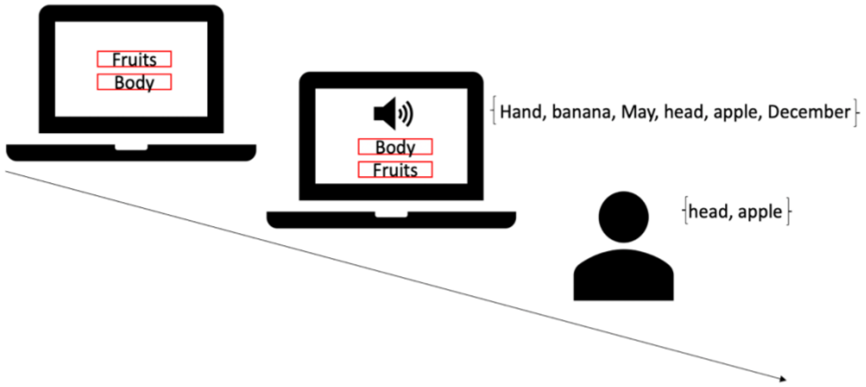

Visuospatial updating task

Like the verbal updating task, this task involved 8 sets of shapes each containing from 6 to 12 shapes. Each shape appeared, one at a time, in randomized positions on 4x4 grids for 600 ms, with an interval of 600 ms between them.

Each set included target shapes and distractor shapes selected from the following: star, circle, square, triangle, rhombus and pentagon. As in the verbal updating task, participants were presented with 2 sets of shapes for each level of increasing difficulty deriving from the greater number of target shapes to monitor while watching the whole set appear one after the other. The number of target shapes ranged from 2 to 5, corresponding to a span level of 2 to 5. For example, a set of 6 shapes consisted of 2 target shapes each appearing in 2 different positions, plus one distractor shape occupying two additional positions on the grid.

The task involved recalling the last position of certain (target) shapes. The target shapes remained visible above the grid throughout the presentation of the set. At the end of the presentation, participants were asked to use the mouse to indicate the last position of the target shapes. Below is an example of the task (span 2, Figure S2).

Figure S2

Example of the Visuospatial updating task showing a trial at span level 2.

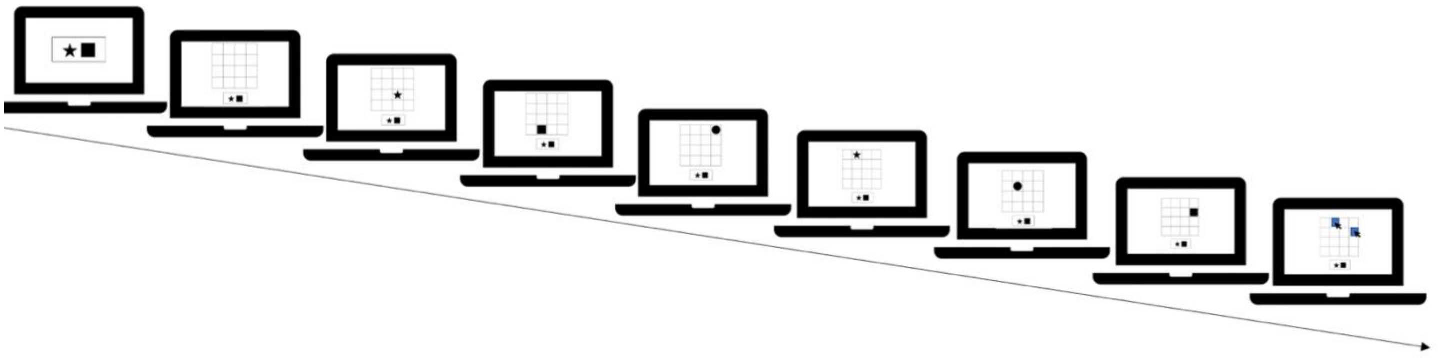

Supplementary Results

Verbal and Visuospatial Updating Span analysis

The accuracy data from the updating tasks were analyzed using generalized mixed-effects models (Baayen, Davidson, & Bates, 2008; Jaeger, 2008) and a "binomial" function family, using the "lme4" package (Bates, Maechler, Bolker, & Walker, 2015). The fixed effects were Group and Span, while Participants and Items were treated as random effects. Participants and Items were included as random effects to consider their variability in each model. The significance of fixed effects was tested through a series of tests for nested models based on the chi-square distribution.

Where necessary, the Benjamini-Hochberg (*BH*) procedure was used to reduce type I error resulting from multiple statistical comparisons (Lesack & Naugler, 2011).

The Akaike information criterion (*AIC*, Akaike, 1974) was also taken into consideration for each of these models. It provided the best description of the relationships between the variables (Bentler, 1990; Schermelleh-Engel, et al., 2003). Graphical effects were obtained using the "effects" package (Fox, 2003).

No main effect of group emerged in the Verbal updating task ($\chi2(3)=6.73$, p=.08, full model: AIC=1815.4, model without Group: AIC=1816.1). Instead, there was a significant main effect of Span ($\chi2(3)=184.52$, p<.001, (model without Span: AIC=1993.9)), suggesting that greater difficulties were encountered with higher Span levels. Specifically, Span level 2 was significantly easier than levels 3, 4 or 5 (p_s <001), and level 3 was significantly easier than level 5 (p=.03). No other significant differences emerged. As shown in Figure S3, there was no significant interaction between Group and Span ($\chi2(9)=4.01$, p=.91, model with Interaction: AIC=1829.4).

In the Visuospatial updating task, in the first stage there was a main effect of Group ($\chi2(3)=10.55$, p=.01, full model: AIC=2013.5, model without Group: AIC=2018). The model coefficients showed that the ADHD and ADHD+SLD groups performed significantly worse than the TD group (p=.01 and p=.007, respectively), with no other significant differences between the groups. The main effect of Span was also significant ($\chi2(3)=100.11$, p<.001, model without Span: AIC=2107.6), suggesting

that participants completed the task with greater difficulty the longer the span. The model coefficients showed that Span level 2 was significantly easier than levels 3, 4 or 5 (p_s<.001), and that level 3 was significantly easier than level 5 (p=.03). No other significant differences emerged.

Finally, the interaction between Group and Span was significant ($\chi 2(9)$=33.63, p<.001, model with Interaction: AIC=1997.8), as shown in Figure S4. Multiple comparisons revealed specific differences: at Span level 3 the TD group performed significantly better than the ADHD (p<.001, Cohen's d=.82), ADHD+SLD (p=.01, Cohen's d=.27), or SLD (p<.001, Cohen's d=.23) groups, and the SLD group performed significantly better than the ADHD (p=.004, Cohen's d=.79) or ADHD+SLD (p=.06, Cohen's d=.51) groups; at Span level 4 differences only emerged between ADHD+SLD and TD, with the former's performance significantly worse than the latter's (p=.03, Cohen's d=.40). As for within-group differences for each Span, each level differed from the others in the TD group (p_s<.001), with the children becoming less accurate the higher the Span level. The ADHD group also performed significantly better on Span level 2 than on level 3 (p<.001), level 4 (p=.009) or level 5 (p=.009), and they fared worse on Span level 3 than on levels 4 (p=.009) or 5 (p=.009). The SLD group's performance was better for Span level 2 than for levels 4 (p=.02) or 5 (p=.006). Finally, the ADHD+SLD performed significantly better on Span level 2 than on levels 3 (p=.009), 4 (p=.006) or 5 (p=.01). No other significant differences emerged.

Figure S3

Predicted accuracy by Group and Span in the Verbal updating task. Error bars represent 95% confidence intervals.

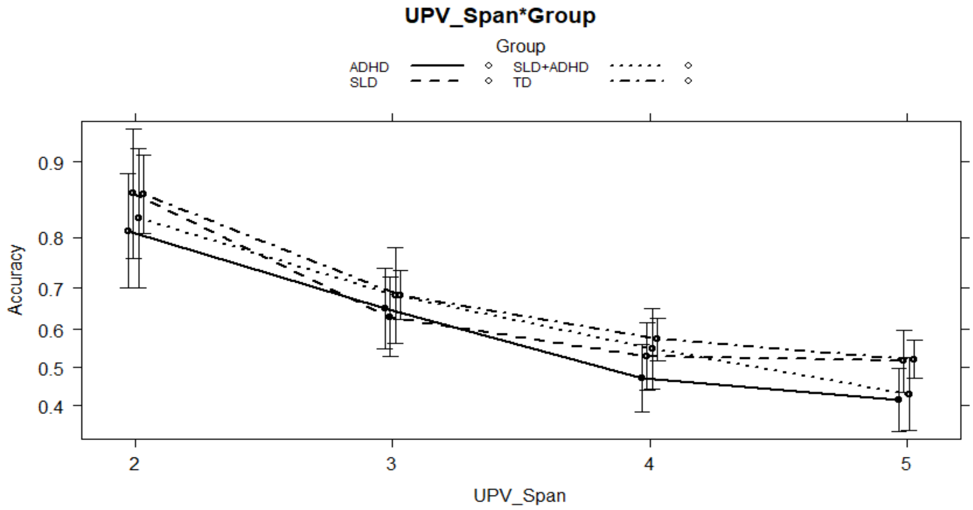

Figure S4

Predicted accuracy by Group and Span in the Visuospatial updating task. Error bars represent 95% confidence intervals.

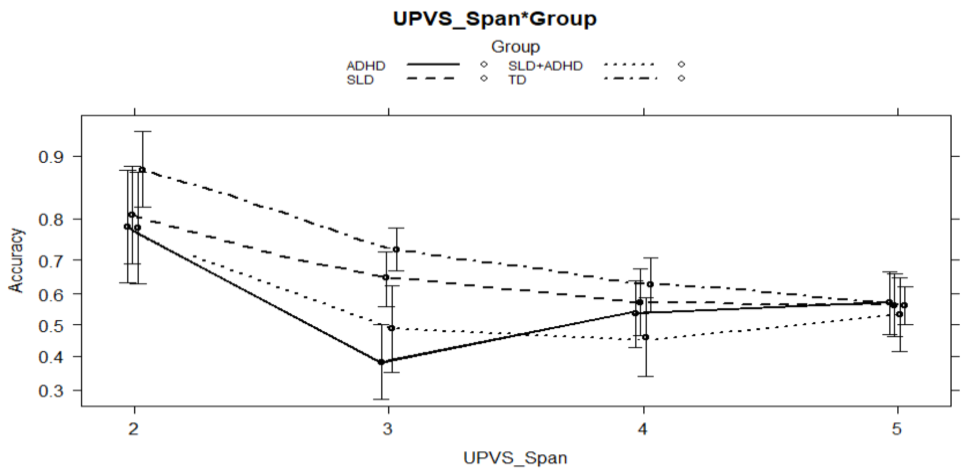

Supplement: Supplementary file 1 [file Table_1.pdf]
